# Sustaining transformative change in public health in Africa to achieve health development goals

**DOI:** 10.1017/S0950268825000123

**Published:** 2025-02-06

**Authors:** Benido Impouma, Lindiwe Makubalo, Kasonde Mwinga, Joseph Cabore, Matshidiso Rebecca Moeti

**Affiliations:** World Health Organization, Regional Office for Africa, Cité de Djoué, Brazzaville, Congo

**Keywords:** communicable and non-communicable diseases, ebola and COVID-19, neglected tropical diseases and SDGs, transformation agenda, WHO African region

## Abstract

In 2015, the WHO African Region was responding to the largest Ebola virus disease outbreak in history while at the same time working to contain a wild poliovirus outbreak [1]. The 2030 Agenda for Sustainable Development had recently been endorsed, reflecting new global development priorities. By 2016, the Ebola outbreak was under control, and a new approach to reform and priority setting was in place in the region; the Transformation Agenda [2]. This agenda, introduced by the new Regional Director for Africa, Dr Matshidiso Moeti, set up a robust system for improving the efficiency and accountability of the WHO Secretariat for the African Region, which has been instrumental in the transformative changes that have been seen across the region in the past 10 years. This commentary discusses significant contributions to public health in the WHO African Region in the past decade, in the context of the Transformation Agenda, and the contributions of major investment in health security in the region. It is important to understand the need to sustain particular initiatives and elements of the transformative change that has taken place in the region.

In 2015, the WHO African Region was responding to the largest Ebola virus disease outbreak in history while at the same time working to contain a wild poliovirus outbreak [1]. The magnitude, geographical spread, and duration of the Ebola virus disease outbreak brought the structure and technical capacity of the WHO secretariat to deliver on its mandate in the African Region into sharp focus. At the same time, the 2030 Agenda for Sustainable Development had recently been endorsed, reflecting new global development priorities. By 2016, the Ebola outbreak was under control. However, the concerns raised during the Ebola virus disease outbreak had prompted the development of a new approach to reform and priority setting in the region. The Transformation Agenda [2], a vision and strategy for change, aimed at facilitating the emergence of the WHO that staff and stakeholders wanted. Set up and introduced by the new Regional Director for Africa, Dr. Matshidiso Moeti, this agenda was formulated after wide consultation with staff within and outside the WHO African Region and stakeholders from outside the organization. The four focus areas of the Transformation Agenda – pro-results values; smart technical focus; responsive strategic operations; and effective communications and partnerships – set up a robust system for improving the efficiency and accountability of the WHO Secretariat for the African Region. This agenda has been instrumental in the transformative changes that have been seen across the region in the past 10 years.

This commentary discusses significant contributions to public health in the WHO African Region in the past decade, in the context of the Transformation Agenda, and the contributions of major investment in health security in the region. It is important to understand the need to sustain particular initiatives and elements of the transformative change that has taken place in the region. At the same time, the exercise of looking backwards can inform priorities moving forward and ensure that the momentum gained in the past decade is not lost. The discussion highlights important investments in health security in the region and the role these played in the COVID-19 response. The main components of this transformative change are made up of a multi-disease elimination agenda leading to a new strategy for ending disease in Africa; leveraging on the successful elimination of major neglected tropical diseases (NTDs) in the region; the importance of leadership, and how this is being harnessed to ensure that women play a major role in controlling NTDs in Africa and the critical importance of the health of women, adolescents, and children as indicators of public health status.

In 1998, the Integrated Disease Surveillance and Response (IDSR) strategy was adopted across the WHO African Region [3], and then further revised in 2010. The region joined the global movement towards improved health security by adopting the revised International Health Regulations 2005 (IHR 2005). The mandate of both IDSR and IHR 2005 has now broadened to cover epidemic-prone diseases, diseases targeted for eradication and elimination, other major diseases, events, or conditions of public health importance and diseases or events of international concern. Looking back, the early 2010s were a period of rapid change for the WHO African Region. Regionally, while most deaths were caused by HIV/AIDS, malaria, and tuberculosis, noncommunicable diseases (NCDs) were already on the rise.

In 2015, NTDs were a significant burden in the region, causing disfigurement, disability, and death among the region’s most vulnerable communities. Today, progress towards the elimination, eradication, and control of these diseases is one of Africa’s greatest success stories, highlighting the regions’ inclusive approach in its policy of leaving-no-one-behind. Since its inception in 2016, WHO African Region, through its Expanded Special Project for Elimination of NTDs (ESPEN) and its partners, have been responsible for the donation and distribution of more than 3.1 billion tablets between 2016 and 2023, with 38 countries reaching 100% geographical coverage over 1 year for at least one NTD [6]. By 2023, 19 countries in the region had eliminated at least one NTD, compared to seven in 2015 – resulting in 88 million fewer people needing health interventions. Four countries have eliminated one of five priority diseases: Malawi and Togo have eliminated lymphatic filariasis, and The Gambia, Ghana, Malawi, and Togo have eliminated trachoma. Togo achieved a world first in 2022 by eliminating four NTDs.

Women, children, and adolescents are a vital part of our communities in the African Region, home to the youngest populations on the globe. The year 2015 marked the end of the Millenium Development Goals, which included targets to reduce under-five mortality by two-thirds between 1990 and 2015 (Goal 4) and other health and nutrition targets important for child health. Several low-income countries in the WHO African Region – Eritrea, Ethiopia, Liberia, Madagascar, Malawi, Mozambique, Niger, Rwanda, Uganda, and the United Republic of Tanzania – recorded a strong reduction in under-five mortality. This showed that low income need not be an impediment to saving children’s lives [7]. From 2016, there was a major push to redress the poor quality of care, recognized as a major driver of poor health outcomes for women and children in the region.

Progress in child survival in the region can be attributed to the sustained commitments of governments, local communities, health workers, and partner organizations. This has led to increased coverage of proven high-impact interventions. These include early initiation of breastfeeding, postnatal care for newborns, early infant HIV diagnosis, exclusive breastfeeding, antibiotics for pneumonia, oral rehydration therapy and zinc for diarrhoea, use of insecticide-treated bed nets, universal access to antiretroviral therapy for HIV, improvements in routine immunization coverage, and introduction of new vaccines, such as rotavirus for childhood diarrhoea.

Continuing the theme of inclusivity and human rights, the past decade has seen the Regional Office focus on gender as a factor in access, outcomes, and agency in health. In the spirit of the Pathways to Leadership for Health Transformation Programme introduced by the Regional Office, the Mwele Malecela Mentorship Programme for Women in NTDs was established in 2022. This programme honours the late Dr. Mwele Malecela, one of Africa’s most respected woman scientists and public health leaders, who served as the Director of the WHO African Region’s Regional Director’s Office and then as Director of NTDs, WHO Headquarters in Geneva. The programme has been established to empower women to emerge as leaders and contribute maximally to NTD interventions. Through this, at least five women each year from 2023 to 2030 will receive mentorship, training, and networking opportunities.

However, the African Region still falls short of aspirations for the health and well-being of children and adolescents. Two major agendas, covering child and adolescent health, have been launched in the past 10 years. In 2017, the Adolescent Health Flagship Programme was launched in conjunction with the Global Accelerated Action for the Health of Adolescents (AA-HA!) guidance. Then, in 2023, the WHO African Region embarked on a multi-step process to co-create a comprehensive agenda for child health in the region, in close cooperation with member states and stakeholders. This agenda aims to drive and accelerate improvement in survival, health, and well-being of children and adolescents. The focus is now on a life-course approach, from 0 to 19 years, including children 5–9 years and adolescents, including pre-conception and pregnancy interventions. Moving beyond a focus on survival will ensure that children and adolescents thrive and can transform their communities. An important component of these interventions is a push to reduce teenage pregnancies [8].

Maternal mortality is another critical indicator of public health status. Although the maternal mortality ratio in the WHO African Region fell by 33.2% between 2000 and 2020, at 531 deaths per 100,000 live births, it is still too high. Increasing access and use of sexual and reproductive health services across the region has been and will continue to be a major contributor to gains in maternal, child, and adolescent health. These services include access to skilled birth attendants and postnatal care and access to modern contraceptive methods. Antenatal care, at least four visits during pregnancy, has also played a role in providing women with appropriate health interventions.

In 2020, Africa joined the rest of the world, in responding to the largest pandemic of the 21st century, COVID-19. This highlighted, globally and regionally, the critical importance of leadership in health, exemplified by the strong political leadership, at the highest levels, shown in the African Region. Rapid action led to the implementation of public health and social measures, as well as unprecedented vaccine rollouts, despite inequality of access, safeguarding African populations against predicted catastrophic levels of morbidity and mortality [4]. However, at the same time, the COVID-19 pandemic exposed continuing underlying weaknesses in health systems across the region. This required a further shift in priority setting across the region, to ensure a move towards agile, equitable, and sustainable health systems and universal health coverage.

Building on the lessons learnt during the early part of the COVID-19 pandemic, a multi-disease elimination agenda – the Ending Disease in Africa (ENDISA) strategy – that aims for an integrated, systems approach to disease prevention, control, elimination, and eradication, was adopted in 2021. The ENDISA strategy aims at supporting countries in renewing and accelerating their efforts to deliver health services and get back on track towards the 2030 SDG targets [5]. Four special initiatives were informed by the core success factors of the COVID-19 pandemic response – governance and system capacity, institutional capacity, data science capacity, and research and innovation capacity, which are being operationalized in the run-up to 2030. ENDISA advocates for health in all policies and strengthened engagement within and beyond the health sector, to address health issues requiring whole-of-government and whole-of-society action.

What are the lessons learnt from this decade of progress and success? How can we use these lessons to move forward and ensure that the young population of the region will enjoy good health, so allowing for economic growth and prosperity? The COVID-19 pandemic and its aftereffects showed the critical importance of leadership and collaboration between political and health actors at the highest levels, regionally and nationally. In addition, two central lessons came out of the pandemic: the importance of Africa’s regional centres of excellence and the critical importance of data for health system strengthening. Collaboration with the region’s universities and other institutions should be expanded and intensified. Equally important are data systems, digital capacity, and innovations in technology. This is all needed to continue to address the urgent health priorities across Africa.

However, Africa cannot act alone. The other major lesson learnt from the pandemic is the importance of equity and collective action across regions and countries, which leads to the ongoing work by the Intergovernmental Negotiating Body on the text of the WHO Pandemic Agreement, aiming to prepare the world to prevent and respond to future pandemics. Consensus has not yet been reached, but the WHO Africa Region will continue to focus on implementing innovative approaches to ensuring the health of its communities by leveraging the huge progress made in responding to outbreaks and emergencies in the past decade [9].

The inequality of access to vital diagnostics, therapeutics and vaccines experienced by Africa during the COVID-19 pandemic demonstrates the importance of regional advances in research, innovation, development, and manufacturing locally. Groupings such as the regulatory body, the African Vaccine Regulatory Forum, the Regional Immunization Technical Advisory Group, and the African Medicines Agency, supported by the WHO Regional Office for Africa, the African Union, and partners will be critical to this expansion. We also learnt during the pandemic that miscommunication and misinformation can impact the uptake of vital vaccines and therapeutics. The Africa Infodemic Response Alliance (AIRA), led by the WHO Regional Office for Africa, was formed by UNICEF, Africa CDC, International Federation of Red Cross and Red Crescent Societies, UNESCO, Verified and UN Global Pulse, to share safe, proven facts to counter dangerous health misinformation.

Another major priority is recognizing and responding to the effects of climate change on pathogen distribution and the resulting changing patterns of disease, and in the large-scale displacement of people, caused by flooding, drought, and food insecurity. This will require a focus not only on emergency response but also on ensuring leadership, partnership, and collaboration to bring a contextual approach to addressing these issues as they arise on the continent. This will require preparedness, resilience, joint global action, and investments in mitigation measures, as well as adaptation to changing global contexts and priorities.

Sustainability of all responses to health priorities in the region must build on a commitment to health system strengthening. Countries in the African Region experience common and cross-cutting issues of health care, especially in the areas of health workforce, health system organization, essential medicines and supplies, access to health facilities, and training in and utilization of technologies and medical equipment. [10] This requires investment in digital health, led by governments and supported by the Regional Office, to strengthen health systems, extend services in hard-to-reach areas and reduce wastage of health resources. These investments will be made in the context of sharply reduced funds and mounting health challenges. The African Region, led by WHO and other major health agencies, should remain agile in its response, with a focus on supporting increased domestic funding, diversifying funding sources and expanding fundraising efforts, while strengthening collaboration with global institutions to pool resources reduce duplications and emphasize shared responsibility for global health goals. This, in turn, will require strengthening advocacy and diplomacy, emphasizing the alignment of WHO initiatives with global security and economic stability. Ensuring return on investment for governments and donors requires that critical programmes are identified and prioritized, while at the same time planning for gradual scaling of activities that may face funding reductions, to ensure minimal disruption to essential health services. As the African Region moves into this challenging world, it will be essential to continue to build regional resilience and sustain funding for priority health programmes and initiatives, contributing to pandemic preparedness and the attainment of health goals.

## Data Availability

The datasets generated during and/or analyzed during the current study are available from the corresponding author upon reasonable request.
